# Integrated Transcriptomic and Metabolomic Analyses of the Response of Lutein Accumulation in Marigold Petals to Light Intensity

**DOI:** 10.3390/genes16111350

**Published:** 2025-11-09

**Authors:** Haimin Zhang, Hong Qiu, Meng Xue, Palinuer Aiwaili

**Affiliations:** College of Horticulture, Xinjiang Agricultural University, Urumqi 830052, China; 15803231864@163.com (H.Z.); 13219981108@163.com (H.Q.); xm030514@163.com (M.X.)

**Keywords:** carotenoids, light intensity, lutein, qRT-PCR, *Tagetes erecta* L., UPLC-MS

## Abstract

[Background] Marigold (*Tagetes erecta* L.) is the main source of the natural pigment lutein. [Methods] In this study, Marigold served as the experimental material for systematic observation of floral organ development. Based on floral morphology and lutein content, the full-flowering stage was identified as the optimal harvesting period. [Results] Under different light intensity gradients (30–1500 μmol·m^−2^·s^−1^), the highest lutein content in petals occurred at ≈500 μmol·m^−2^·s^−1^. Increased light intensities promoted flowering and enlarged flower diameter while significantly shortening the growth cycle. Transcriptome analysis revealed that light intensity variation markedly influenced the expression of genes related to metabolic pathways, plant hormone signal transduction, and carotenoid biosynthesis, and enriched transcription factor families including bHLH, MYB, NAC, and WRKY. Metabolomic profiling identified lutein esters, such as lutein dimyristate and lutein dipalmitate, as the dominant accumulated forms, with their contents positively correlated with light intensity; under high light, intermediate metabolites, including α-cryptoxanthin and zeaxanthin, were significantly up-regulated. [Conclusions] This study clarifies the molecular mechanism by which light intensity precisely regulates lutein accumulation through coordinated synthesis, esterification, and degradation pathways, offering a theoretical foundation for light-regulated cultivation of *T. erecta* L. and efficient lutein production.

## 1. Introduction

Marigold (*T. erecta* L.), an annual herbaceous plant belonging to the Asteraceae family [1,2]. Native to Mexico and the Americas, it is now widely distributed in the United States, Latin America, Asia, and other regions [3]. With numerous cultivated varieties, marigold not only possesses high ornamental value but also serve as a natural plant-derived dye [4]. The orange petals of marigolds are rich in carotenoids, with lutein accounting for approximately 90% of the total carotenoid content. This makes marigolds one of the most abundant natural sources of lutein [4].

Lutein (β,ε-carotene-3,3′-diol) is primarily synthesized in plants, and humans cannot synthesize lutein to meet physiological demands, necessitating its extraction from natural sources [5]. Lutein and its isomers play critical roles in ocular development throughout the fetal life cycle, visual performance during childhood and adulthood, and the reduction in age-related ocular disease risks in elderly populations [6]. Additionally, lutein protects the human body against various free radical damages, including photoprotection for the skin and eyes [7], while exhibiting both anticancer activity and cardiovascular protective effects [8].

The biosynthesis of carotenoids begins with the condensation of two molecules of the precursor geranylgeranyl diphosphate (GGPP) by phytoene synthase (PSY), forming phytoene. PSY is widely recognized as a key rate-limiting enzyme in the carotenoid pathway, and its activity and expression level often determine the total flux into the pathway [9]. Subsequently, phytoene is converted into lycopene through a series of enzymatic reactions catalyzed by phytoene desaturase (PDS), ζ-carotene desaturase (ZDS), and carotenoid isomerase (CrtISO), with ζ-carotene as an intermediate [10]. Lycopene is then cyclized at either the β- or ε-position by lycopene β-cyclase (β-LCY) and lycopene ε-cyclase (ε-LCY), respectively, bifurcating the carotenoid pathway into β- and α-branches. In the β-branch, β-LCY catalyzes the formation of β-rings at both ends of lycopene, yielding γ-carotene as an intermediate, which is further converted into β-carotene. Subsequently, β-carotene hydroxylase (β-OHase) mediates the hydroxylation of β-carotene via β-cryptoxanthin, ultimately producing zeaxanthin. In the α-branch, ε-LCY first cyclizes one end of lycopene to form δ-carotene with a δ-ring, followed by β-LCY-mediated cyclization at the other end to generate α-carotene. α-Carotene is then hydroxylated by carotenoid ε-ring hydroxylase (ε-OHase) to form α-cryptoxanthin, followed by further hydroxylation via β-OHase, resulting in the synthesis of lutein [11,12]. Ultimately, carotenoids are degraded via carotenoid cleavage dioxygenases (CCDs), xanthoxin dehydrogenase [ABA DEFICIENT 2 (ABA2)], 9-cis-epoxycarotenoid dioxygenases (NCEDs), abscisic aldehyde oxidase 3 (AAO3), and other enzymes [13,14].

Lutein, a carotenoid, serves as a photosynthetic pigment that functions in light energy harvesting and transfer, while also protecting chlorophyll from excessive light exposure. Light intensity significantly influences the lutein cycle. Under high-light conditions, the biosynthesis of zeaxanthin and neoxanthin is enhanced, facilitating the light-harvesting complexes (LHCs) in dissipating excess light energy and thereby safeguarding plant tissues from photodamage [15,16]. In studies on *Chlamydomonas* sp., exposure to high-intensity light has been shown to reduce the content of most carotenoids and chlorophyll, along with decreased lutein levels and downregulation of the lutein synthesis-related gene *Lut1*, while the concentrations of zeaxanthin and antheraxanthin increased [17]. In a study on the marine microalga *Tetraselmis* sp. CTP4, it was observed that β-carotene content increased threefold under low light intensity (33 μmol m^−2^·s^−1^), whereas lutein levels rose by 1.5-fold under high light conditions (170 and 280 μmol m^−2^·s^−1^) [18]. Recently, in Lettuce (*Lactuca sativa*), elevated light intensity induced a significant increase in the content of all measured carotenoids—including violaxanthin, neoxanthin, zeaxanthin, trans-zeaxanthin, lutein, and α-carotene—as well as total carotenoid content [19]. These findings in lettuce are consistent with observations in marigold, where light intensity is a major environmental factor influencing flower quality and carotenoid profiles, underscoring the complex interplay between light and secondary metabolism in horticultural plants [20]. In summary, due to differences between algae and plants and variations in light intensity settings, conflicting conclusions have been reported in related studies regarding the effect of light intensity on lutein content.

In marigold cultivation, plants experience diverse light environments, ranging from full sunlight in open fields to low light under forest intercropping. The regulatory mechanisms through which light intensity governs lutein accumulation, a core value component, remain poorly understood. This study investigates how light intensity regulates lutein levels in marigolds and tests the central hypothesis of a non-monotonic response, where accumulation initially increases but then declines under higher intensities. We propose that an optimal light intensity exists; below this threshold, insufficient energy limits synthesis, while above it, light stress activates the lutein cycle, converting lutein to zeaxanthin faster than its synthesis and reducing net accumulation.

To evaluate this, we applied a series of light intensity treatments during the peak lutein accumulation period. By combining metabolomics for precise quantification of lutein and its derivatives with transcriptomics for systematic analysis of key synthesis and degradation enzyme genes, this work aims to reveal the light-regulated mechanisms controlling lutein accumulation. Furthermore, understanding the esterification process, which enhances the stability and accumulation of lutein in petal tissues, is crucial for optimizing extraction yields [21]. These findings will establish a theoretical foundation for optimizing cultivation practices to increase the yield of this natural pigment.

Based on the established research framework and hypotheses, we propose three falsifiable biological predictions that can be directly tested with omics data:

Prediction 1: Predominance of Esterification

We hypothesize that esterification becomes the dominant process for lutein storage under light intensities optimal for its maximal accumulation. Metabolomic data should therefore show a peak in the ratio of lutein esters, such as lutein dipalmitate, to free lutein. Corresponding transcriptomic data should reveal a strong positive correlation between the expression of petal-specific ester synthase genes and the total lutein content.

Prediction 2: Transcriptional Regulatory Mechanism

We further hypothesize that light intensity mediates this metabolic reprogramming by activating specific transcription factor families. Transcriptome analyses should show significant differential expression of members from families like bZIP, MYB, or NAC, which are known to integrate light signals with metabolic pathways. Their expression patterns should also co-vary strongly with those of key structural genes in lutein biosynthesis, such as *PSY* and *ε-LCY*, positioning them as potential hub nodes within the regulatory network.

## 2. Materials and Methods

### 2.1. Plant Materials and Light Intensity Treatment

The study employed the widely cultivated marigold (*T. erecta* L.) variety ‘Pigment No. 1,’ which is prevalent in southern Xinjiang. All experiments were conducted at Xinjiang Agricultural University, located at 43.4826° N and 87.343° E. Marigold seeds were sown and then transplanted into 19 cm diameter plastic pots after the seedlings developed 2 to 3 pairs of true leaves. The substrate consisted of a 1:1 mixture of peat and vermiculite, with environmental conditions maintained at 26 ± 1 °C, 40% humidity, and a 16 h light/8 h dark photoperiod. Plants were watered when the soil surface dried and fertilized every 15 ± 2 days. A gradient of light intensity treatments was established as follows: T1 at ≈30 μmol m^−2^ s^−1^, T2 at ≈50 μmol m^−2^ s^−1^, T3 at ≈100 μmol m^−2^ s^−1^, T4 at ≈500 μmol m^−2^ s^−1^, T5 at ≈1000 μmol m^−2^ s^−1^, and T6 at ≈1500 μmol m^−2^ s^−1^. Eight well-growing and uniformly shaped replicate samples were set up for each treatment group for the experiment.

### 2.2. Paraffin Section

Terminal buds from marigold plants at various developmental stages were collected under natural light, with three replicates established for the analysis. The outer leaves were removed, and a fixative solution was prepared containing 5 mL of 38% formaldehyde, 5 mL of glacial acetic acid, 90 mL of 70% ethanol, and 5 mL of glycerol. This solution was added to a centrifuge tube containing the buds, which was sealed and fixed for 24 h. The samples were dehydrated through a graded alcohol series of 50%, 70%, 80%, and 95% ethanol. They were then treated sequentially with mixtures of anhydrous ethanol and xylene in ratios of 2:1, 1:1, and 1:2, followed by two 30 min treatments in pure xylene. After embedding, the wax block was removed from the embedding box, labeled with the corresponding sample number, and sectioned at 10 microns using a microtome. The resulting sections were mounted on clean glass slides and stained using the Saffron-fast green method. Finally, the prepared paraffin sections were observed and photographed under an imaging microscope.

### 2.3. RNA Extraction and Quantitative Real-Time PCR

The tips of the sixth expanded leaves were collected from each of the five replicates per sample at 11:30. Total RNA was extracted from each sample with RNAiso Plus reagent (TaKaRa, Shiga, Japan) following the manufacturer’s protocol. Subsequently, 1 mg of total RNA was reverse-transcribed into cDNA using the HiScript II Q Select RT SuperMix for qPCR (+gDNA wiper) kit (Vazyme, Nanjing, China). Quantitative RT-PCR was then performed in a 20-μL reaction volume containing 2 μL of cDNA as template, employing the StepOne Real-Time PCR System (Sangon, Shanghai, China) in standard mode with the 2’ Realtime PCR Super Mix (SYBRgreen, with anti-Taq). *TeTIF6* was used as the reference gene. The marigold *TeTIF6* gene served as the internal control [22]. The qRT-PCR primer sequences are provided in Supplementary Table S1. Relative expression levels were determined using the 2^−ΔΔCT^ method [23].

### 2.4. Determination of Total Lutein Content

The total lutein content was measured using ultraviolet-visible spectrophotometry. Marigold flower tissue samples at various developmental stages were dried at 60 °C in an electrothermal constant temperature blast drying oven and then ground into a fine powder. Each developmental stage was repeated three times. Samples weighing between 0.10 and 0.15 g were accurately measured using a 1/10,000 balance and placed in a 100 mL brown volumetric flask. To this, 30 mL of an extraction solvent (n-hexane: acetone: toluene: absolute ethanol in a ratio of 10:7:7:6) and 2 mL of a methanol solution containing 35% KOH were added. The flask was shaken in an ultrasonic cleaner for 40 s and then saponified in a water bath at 50 °C for 20 min. After saponification, the sample was allowed to cool to room temperature. Subsequently, 30 mL of n-hexane was added, and the mixture was diluted to 100 mL with a 10% anhydrous sodium sulfate solution. After shaking for 1 min, the solution was left in the dark for 1 h to facilitate chromatographic separation. The volume of the upper clear liquid after standing represented the total lutein extract from the marigold (50 mL). One milliliter of the supernatant was transferred to a 25 mL volumetric flask, and the total lutein eluent (n-hexane) was diluted to create the diluted total lutein extract. The sample solution was then placed in a 1 cm cuvette, and the absorbance at 474 nm was measured using an ultraviolet-visible spectrophotometer.

The equation for calculating the total lutein content is expressed as:

W0 = A × V/(236 × m)(1)

In Formula (1), W0 denotes the total lutein content in grams per kilogram, A indicates the absorbance of the sample solution at 474 nm, V represents the dilution factor, m is the mass of the sample in grams, and 236 is the absorption coefficient for a total lutein sample solution with a mass concentration of 1 g/L at 474 nm in n-hexane.

### 2.5. Transcriptome Analysis

At the S3 stage, the sixth leaf and first flower of marigold under different light treatments were collected (set three repetitions); 1.0 g of petal tissue was weighed on a 1/10,000 balance and immediately frozen in liquid nitrogen. Metware Biotechnology Co., Ltd. (Wuhan, China) conducted cDNA library sequencing on the MGI platform. Raw reads were processed with fastp to remove adapter sequences: paired-end reads were discarded if the number of N bases in either read exceeded 10% of its length, or if low-quality bases (Q ≤ 20) constituted more than 50% of the read length. All downstream analyses utilized the resulting clean reads. Differential gene expression between sample groups was assessed with DESeq2, and *p*-values were adjusted using the Benjamini–Hochberg procedure. Statistically significant differential expression was determined by applying thresholds to the adjusted *p*-values and log_2_ fold changes. Functional enrichment analysis employed the hypergeometric test, with KEGG pathways and GO terms evaluated separately.

### 2.6. Targeted Metabolomic Analysis of Carotenoids

Carotenoid contents were quantified by MetWare (http://www.metware.cn/) using an AB Sciex QTRAP 6500 LC-MS/MS platform. HPLC-grade methanol, ethanol, and acetonitrile were obtained from Merck (Darmstadt, Germany), while BHT was sourced from Aladdin. Acetone was purchased from Sinopharm, methyl tert-butyl ether from CNW, sodium chloride from Rhawn, and potassium hydroxide from Hushi. All experiments utilized Milli-Q water from Millipore (Bradford, PA, USA). Standards were acquired from Sigma-Aldrich (St Louis, MO, USA) and BOC (NY, USA), with formic acid also supplied by Sigma-Aldrich. Stock solutions of the standards were prepared at 1 mg/mL in MTBE/MeOH and stored at −20 °C.

Samples were freeze-dried, pulverized (30 Hz, 1.5 min), and stored at −80 °C until analysis (set three repetitions). A 50 mg aliquot of the powder was extracted with 0.5 mL of an n-hexane/acetone/ethanol mixture (1:1:1, *v*/*v*/*v*). The mixture was vortexed for 20 min at room temperature, then centrifuged at 12,000 r/min for 5 min at 4 °C to collect the supernatant. The residue was re-extracted following the same procedure. The combined supernatants were evaporated to dryness and reconstituted in 150 μL of dichloromethane. Finally, the solution was filtered through a 0.22 μm membrane for LC-MS/MS analysis [24,25,26].

Sample extracts were analyzed using a UPLC-APCI-MS/MS system (UPLC, ExionLC™ AD, https://sciex.com.cn/; MS, Applied Biosystems 6500 Triple Quadrupole, https://sciex.com.cn/). Chromatographic separation was performed on a YMC C30 column (3 μm, 100 mm × 2.0 mm i.d.) with a mobile phase consisting of methanol/acetonitrile (1:3, *v*/*v*) containing 0.01% BHT and 0.1% formic acid (solvent A) and methyl tert-butyl ether with 0.01% BHT (solvent B). The gradient program began at 0% B (0–3 min), increased to 70% B (3–5 min), then to 95% B (5–9 min), and returned to 0% B (10–11 min). The flow rate was 0.8 mL/min, the column temperature was maintained at 28 °C, and the injection volume was 2 μL [24,27].

### 2.7. Transcriptomic and Metabolomic Integrative Analysis

The Pearson correlation coefficient (R^2^ > 0.9) was used to assess the correlation between DEGs and DAMs across the comparisons of Treat CK, Treat 500, and Treat 1500. The resulting network was visualized using Cytoscape software (v 3.7.2).

## 3. Results

### 3.1. Flower Organ Development of Marigold

**Figure 1:**
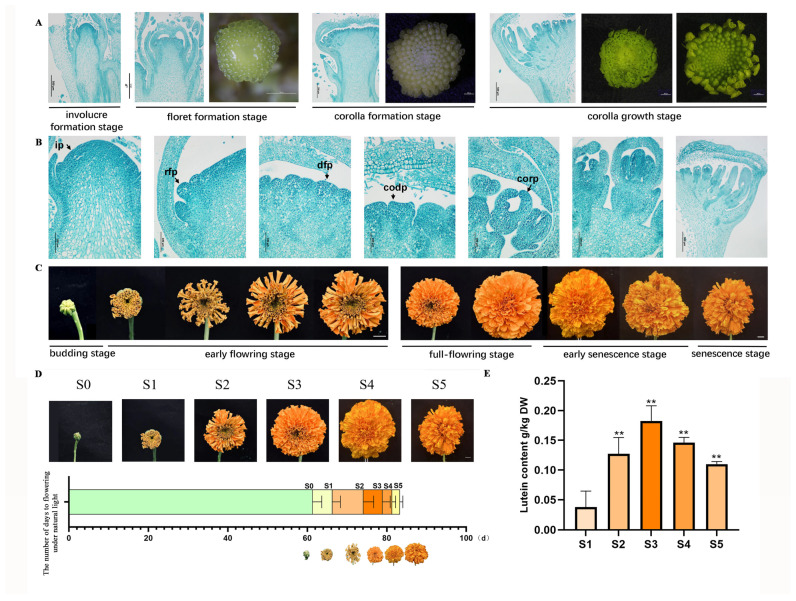
The developmental stages of marigold flower organs and the determination of lutein content. (**A**,**B**) Observation of flower buds at different developmental stages of marigold by a stereomicroscope and paraffin cross-section. ip: inflorescence primordium; rfp: ray floret primordium; dfp: disk floret primordium; codp: corolla primordium of disk floret; corp: corolla primordium of ray floret. Bars = 100 μm. (**C**) Morphological observation of flower buds at different developmental stages of marigold. Bars = 1 cm. (**D**) The time of different developmental stages of marigold. (**E**) Lutein content in marigolds at different developmental stages. Three independent experiments were performed, and error bars indicate standard deviations. Significant differences were determined by *t*-test (** *p* < 0.01).

Marigold (*T. erecta* L.), as a member of the Asteraceae family, possesses the characteristic inflorescence type of this family—the capitulum. Anatomical observation of marigold floral organs revealed that their development follows the developmental pattern typical of Asteraceae species. It begins with the involucre formation stage, followed sequentially by the initiation of floret formation stage at the outermost whorl, corolla formation stage, and corolla growth stage, progressing acropetally from the periphery toward the center (Figure 1A). After the floral transition, floret primordia differentiate starting from the outermost region of the involucre. Throughout the developmental process from floret primordia emergence to corolla elongation, the petals of the ray florets undergo continuous elongation (Figure 1B). Following the formation of macroscopically visible flower buds, marigold flowers progress through several stages: the budding stage, early flowering stage, full-flowering stage, early senescence stage, and senescence stage (Figure 1C).

### 3.2. Marigolds Reached Their Highest Lutein Content at the Full-Flowering Stage, S3

This study divided the marigold flowering process into six distinct stages. Seedlings were transplanted into large pots after the fourth leaf emerged under natural light conditions. Transplantation to the bud stage (S0) required 60 ± 1 days. The petal coloration stage (S1) occurred after an additional 2 ± 1 days. A further 5 ± 1 days were needed to reach the initial flowering stage (S2), and another 8 ± 1 days elapsed before the full-flowering stage (S3). The early senescence stage (S4) began 5 ± 1 days after full bloom, and the flower senescence stage (S5) was observed 2 ± 1 days later. The total period from transplantation to the end of flowering spanned 80 ± 1 days (Figure 1D). Measurements of petal lutein content across these stages showed that the level at S3 was significantly greater than at any other stage, identifying S3 as the optimal harvest time for marigold (Figure 1E).

### 3.3. Expression Levels of Lutein Biosynthesis Pathway Genes at Different Developmental Stages of Marigold

**Figure 2:**
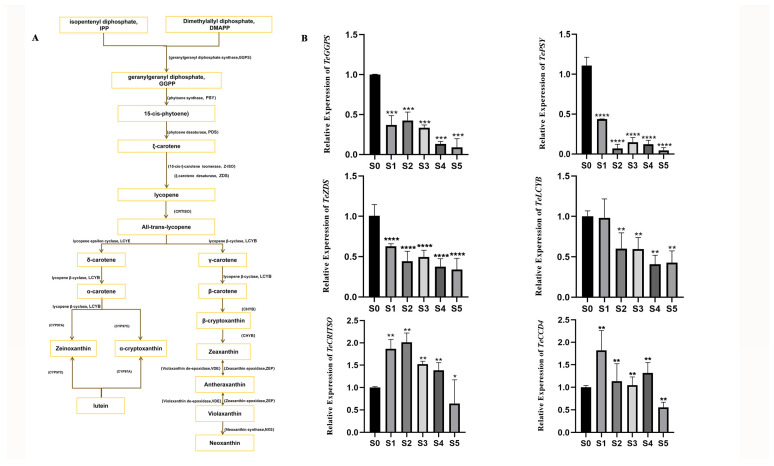
Expression patterns of key genes in the lutein synthesis pathway were detected across different developmental stages of mariglod. (**A**) Marigold lutein metabolic pathway. (**B**) Three independent experiments were performed, and error bars indicate standard deviations. Significant differences were determined by *t*-test (* *p* < 0.1; ** *p* < 0.01; *** *p* < 0.001; **** *p* < 0.0001).

The lutein biosynthesis pathway is depicted in Figure 2A. To characterize the expression dynamics of key genes in this pathway, their transcript levels were quantified across different developmental stages of marigold flowers. As shown in Figure 2B, the genes *TeGGPS*, *TePSY*, and *TeZDS* were most highly expressed prior to flowering, with peak levels occurring at stage S0. Expression of the *TeLcyb* gene remained relatively high at stages S0 and S1. In contrast, *TeCRITSO*, which acts late in the pathway, showed stronger expression during the flowering period and reached its maximum at stage S1. We also assessed the expression of *TeCCD4*, a central gene in carotenoid degradation. *TeCCD4* transcript levels increased during flowering and peaked at stage S1 (Figure 2B).

### 3.4. Determination of Lutein Content Under Different Light Intensity Treatments

**Figure 3:**
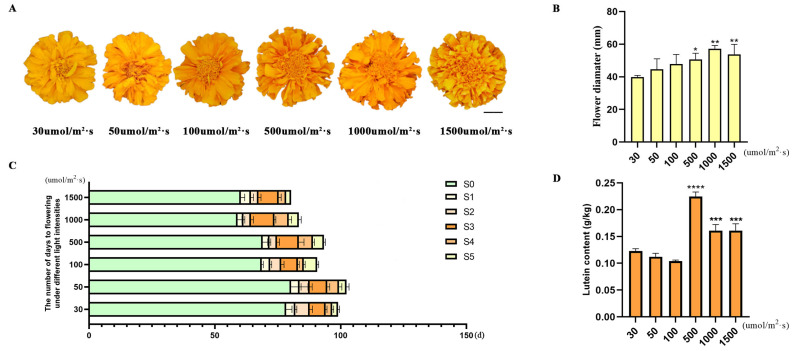
The phenotypes and lutein content of marigolds under different light intensity treatments. (**A**) Flower morphology observation of marigold at S3 stage under different light conditions; Bars = 1 cm. (**B**) Flower diameter of marigold at S3 stage under different light conditions; Significant differences were determined by *t*-test (* *p* < 0.05; ** *p* < 0.01). (**C**) The flowering time of marigold under different light conditions; (**D**) Lutein content in petals of marigold at S3 stage under different light conditions; Three independent experiments were performed, and error bars indicate standard deviations. Significant differences were determined by *t*-test (*** *p* < 0.001; **** *p* < 0.0001).

Different light intensity gradients were established to examine changes in lutein content within marigold petals. Marigold plants with a height of 28 ± 3 cm were exposed to light intensities of ≈30, 50, 100, 500, 1000, and 1500 μmol/m^2^·s until flowering (Figure 3A). These varying light conditions produced flower color phenotypes of differing intensities (Figure 3A). Flower diameter was measured at the S3 stage post-anthesis, revealing a positive correlation with increasing light intensity (Figure 3B). The flowering progression was also analyzed under the different light regimes. Higher light intensities shortened the time required to reach the bud stage (bud diameter of 4 ± 1 mm) and accelerated the overall flowering process. At ≈30 μmol/m^2^·s, marigolds required 106 ± 1 days to reach the senescence stage, whereas at ≈1500 μmol/m^2^·s, this period decreased to 80 ± 1 days, a difference of 26 days. The total flowering period duration was also reduced, although not significantly (Figure 3C). Petals collected at the S3 stage from each light intensity condition were used for lutein extraction and content measurement. Lutein content was generally higher in the three groups exposed to greater light intensities compared to those under lower light. The maximum lutein content of 0.22 g/kg was recorded under the ≈500 μmol/m^2^·s light intensity condition (Figure 3D).

### 3.5. Transcriptome Analysis of Lutein Response to Different Light Intensities

#### 3.5.1. Overview of Transcriptome Data

**Figure 4:**
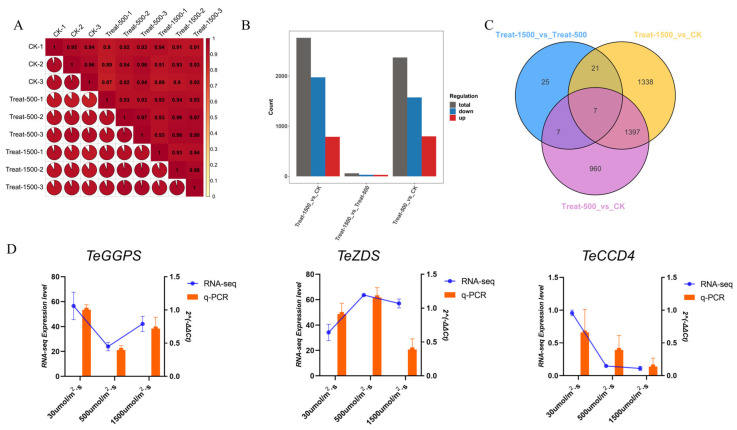
Overview of transcriptome data of mariglod in light intensity stress. (**A**) Sample correlation chart; (**B**) The number of up-regulated and down-regulated DEGs in different light intensity treatments; (**C**) Venn diagram of DEGs treated with different light intensity treatments; (**D**) The expression patterns of three genes under different light treatment conditions were cross-validated by RNA-seq and qRT-PCR; Three independent experiments were performed, and error bars indicate standard deviations.

Transcriptome analysis was conducted on marigold petals exposed to different light treatments. Based on preliminary studies, petals at the S3 stage under weak light (≈30 μmol m^−2^·s^−1^; control), optimal light for lutein content (≈500 μmol m^−2^·s^−1^), and high light (≈1500 μmol m^−2^·s^−1^) conditions were selected for transcriptome sequencing. Nine cDNA libraries were constructed and sequenced using the Illumina platform, yielding a total of 70.61 Gb of clean data, with each sample providing at least 5 Gb. The Q30 base percentage reached 96% or higher. The reference genome GCA_030867185.1_ASM3086718v1_genomic.fna was used for this project, and over 90% of the sequencing reads from the transcriptome samples mapped successfully to the genome (Supplementary Table S2). Sample correlation analysis showed coefficients close to 1 among replicates within the same group (Figure 4A), confirming that all three biological replicates for each sample were suitable for further analysis.

Using low light conditions as the control, we established three comparison groups: CK vs. Treat500, CK vs. Treat1500, and Treat500 vs. Treat1500. These comparisons identified 2371 (1574 up-regulated and 797 down-regulated), 2763 (1975 up-regulated and 788 down-regulated), and 60 (31 up-regulated and 29 down-regulated) differentially expressed genes (DEGs), respectively. The CK vs. Treat1500 comparison exhibited the highest number of DEGs, whereas very few DEGs appeared between Treat500 and Treat1500. In all comparisons, the number of up-regulated genes surpassed that of down-regulated genes (Figure 4B). Among the three groups, 960, 1338, and 25 DEGs were unique to each comparison, respectively. Because the Treat500 vs. Treat1500 group contained so few DEGs, only 7 were common to all three comparisons. These 7 conserved DEGs were primarily associated with molecular chaperones, specifically the small heat-shock proteins Hsp26 and Hsp42 (Figure 4C). Combined with the floral phenotypes observed under different light conditions, substantial phenotypic differences and considerable variations in lutein content were evident between low and high light intensities. The transcriptome data, reflected in the number of DEGs, further confirmed that phenotypic differences between 500 μmol/m^2^·s and 1500 μmol/m^2^·s were minimal. To further validate the reliability of the transcriptome data, we analyzed key genes in the lutein biosynthesis pathway using Real-time fluorescence quantitative PCR (qRT-PCR). As shown in Figure 4D, the expression trends from the transcriptome data aligned with those from qRT-PCR, confirming the reliability of the transcriptome data.

#### 3.5.2. DEGs Enrichment Analysis

**Figure 5:**
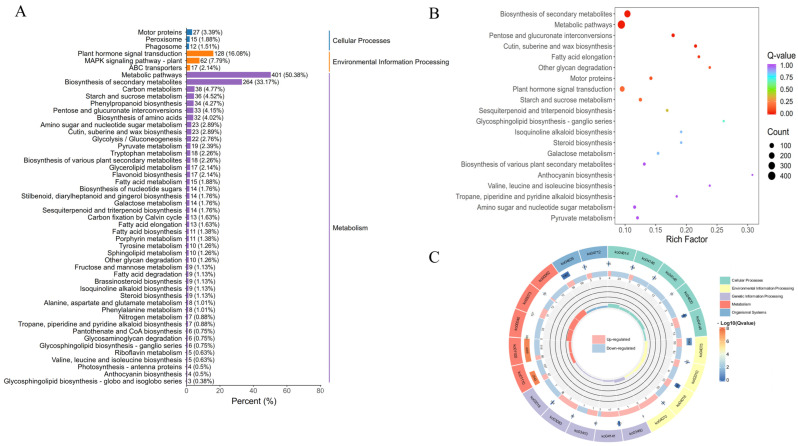
Bar chart for the enrichment of DEGs and KEGG circle plot. (**A**) KEGG number statistical chart; (**B**) KEGG significant bubble diagram; (**C**) KEGG circular plot of differentially expressed genes.

To explore the physiological mechanisms of lutein accumulation in marigold petals under varying light conditions, we performed KEGG analysis on the identified differentially expressed genes (DEGs). These DEGs were annotated to pathways involved in Cellular Processes, Environmental Information Processing, and Metabolism. As illustrated in Figure 5A, most DEGs were enriched within Metabolism in Metabolic pathways and Biosynthesis of secondary metabolites, and within Environmental Information Processing in Plant hormone signal transduction and MAPK signaling pathway—plant. Significant enrichment was particularly evident in Metabolic pathways, Biosynthesis of secondary metabolites, and Plant hormone signal transduction (Figure 5B). In the KEGG enrichment circle plot, five categories related to metabolic pathways showed significant enrichment. Among these, Metabolic pathways and Biosynthesis of secondary metabolites contained the highest numbers of background genes, with 4269 and 2543 entries, respectively (Figure 5C). Both the Metabolic pathways and the Biosynthesis of secondary metabolites pathways also incorporated the carotenoid biosynthesis and zeatin biosynthesis pathways (Supplementary Figures S2 and S3).

**Figure 6:**
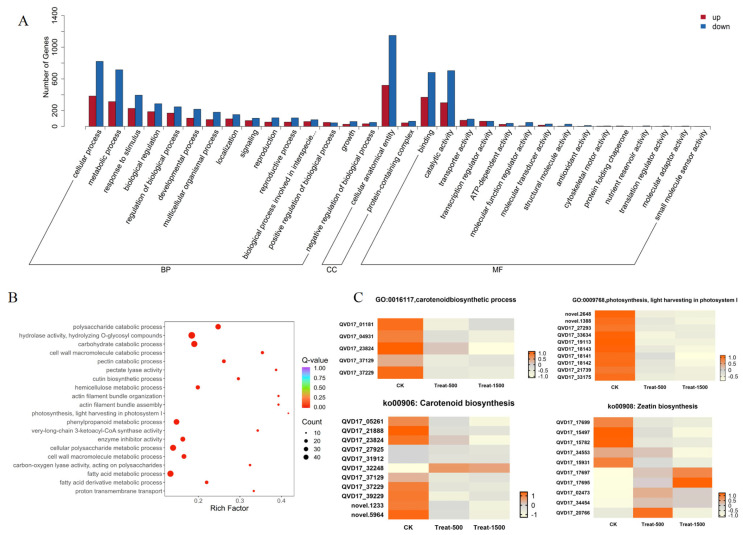
GO classification bar chart and GO enrichment result scatter plot. (**A**) Bar chart of up-regulated and down-regulated genes in GO analysis; (**B**) Scatter plot of GO enrichment results; (**C**) Heatmap analysis of DEGs in significantly enriched pathways of Light intensity treatment in marigold.

We further conducted Gene Ontology (GO) analysis on the DEGs, classifying them into Biological Process, Cellular Component, and Molecular Function. According to the GO classification statistics of up- and down-regulated DEGs, cellular process and metabolic process were predominant in Biological Process, while cellular anatomical entity was the main category in Cellular Component. For Molecular Function, binding and catalytic activity represented the major functional groups (Figure 6A). As shown in Figure 6B, within Biological Process, DEGs were most highly enriched in photosynthesis, light harvesting in photosystem I (GO:0009768), aligning with the altered light intensities in our experiment. In Molecular Function, the highest enrichment occurred for pectate lyase activity (GO:0030570). The most significantly enriched terms were hydrolase activity, hydrolyzing O-glycosyl compounds (GO:0004553) in Molecular Function and carbohydrate catabolic process (GO:0016052) in Biological Process.

Analysis of differentially expressed genes (DEGs) identified significant expression changes across various treatments and pathways in both GO annotation and KEGG classifications. The GO term metabolic process and the KEGG pathway Metabolic pathway both showed significant enrichment. Integrating these analyses revealed that genes co-classified into carotenoid-related pathways under the metabolic process term exhibited pronounced expression changes, as depicted in Figure 6C. Genes annotated with GO:0016117 (carotenoid biosynthetic process) were uniformly down-regulated under high-light conditions. Similarly, genes associated with GO:0009768 (photosynthesis, light harvesting in photosystem I) also displayed downregulation. Within the KEGG pathway ko00906 (Carotenoid biosynthesis), most genes were down-regulated except for gene QVD17_32248, which was up-regulated. In the KEGG pathway ko00908 (Zeatin biosynthesis), genes QVD17_17697, QVD17_17695, QVD17_02473, QVD17_34454, and QVD17_20766 were up-regulated, while genes QVD17_17699, QVD17_15497, QVD17_15782, QVD17_34553, and QVD17_15931 were down-regulated. The up-regulated genes were all identified as UDP-glucuronosyl and UDP-glucosyl transferases, which participate in lutein modification.

#### 3.5.3. Transcription Factor Analysis of Differentially Expressed Genes

**Figure 7:**
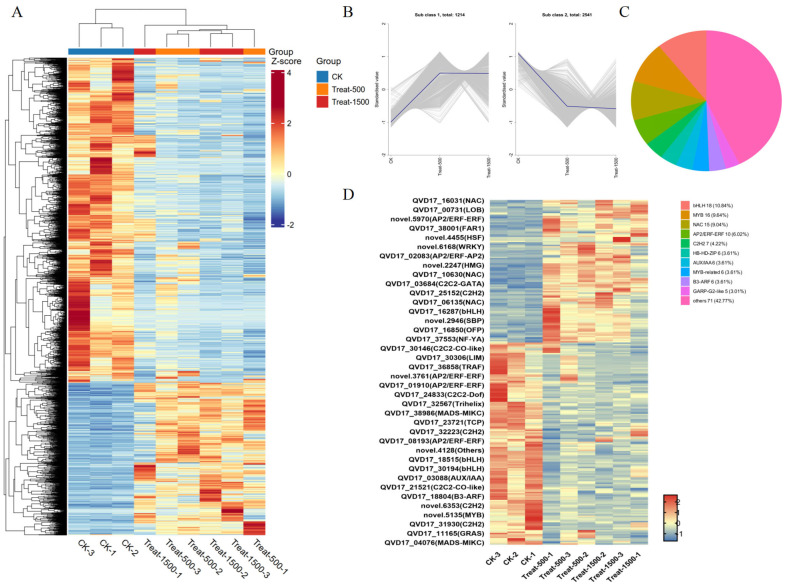
Heatmap of differentially expressed genes and differentially expressed transcription factors. (**A**) Hierarchical clustering heat map for differentially expressed genes; (**B**) K-mean analysis of common differentially expressed genes; (**C**) Pie chart showing the distribution of differential transcription factor gene families (Treat-500_vs_Ck); (**D**) Heatmap of clustered differential transcription factors.

Cluster analysis identified a total of 3755 differentially expressed genes (DEGs). A hierarchical clustering heatmap of these DEGs (Figure 7A) showed similar expression patterns between the 500 μmol/m^2^·s and 1500 μmol/m^2^·s light intensity treatments. Among the DEGs, 1214 genes were up-regulated from low to high light intensity, whereas 2541 genes were down-regulated (Figure 7B). Within this set, 262 genes were annotated as transcription factors (TFs), which are proteins that bind specific DNA sequences to regulate gene expression and play critical roles in development and stress responses. The TFs responsive to light intensity changes were primarily from the bHLH, MYB, and NAC families, with additional members from the AP2/ERF-ERF, C2H2, HB-HD-ZIP, and AUX/IAA families (Figure 7C). As shown in Figure 7D, most of these TFs were down-regulated. Among the up-regulated TFs, WRKY transcription factors were also present, in addition to those already mentioned. According to annotations, novel.6168 was identified as *WRKY24*. WRKY transcription factors can dynamically regulate carotenoid metabolism by activating biosynthetic genes such as *PSY*, *PDS*, and *LCYB*, or by inhibiting catabolic genes like *CCD1* in response to environmental signals. However, no reports have described *WRKY24* functioning in this specific context.

### 3.6. Metabolomic Analysis of Lutein in Response to Different Light Intensities

To investigate the effects of light intensity on lutein synthesis and metabolism, this study conducted a targeted metabolomic analysis of carotenoids. Initially, 72 carotenoid metabolites were detected using liquid chromatography-tandem mass spectrometry (LC-MS/MS). The data acquisition system primarily consisted of an ultra-performance liquid chromatography (UPLC) system (ExionLC™ AD, https://sciex.com.cn/) coupled with tandem mass spectrometry (MS/MS). Qualitative analysis of the mass spectrometry data was performed based on the Metware Database (MWDB) constructed using reference standards. Quantification was achieved through multiple reaction monitoring (MRM) mode on a triple quadrupole mass spectrometer. Mass spectrometry data were processed using Analyst 1.6.3 software. The total ion chromatogram (TIC) and extracted ion chromatogram (XIC) are provided in Supplementary Figures S3 and S4, respectively. Mass spectrometry data were further processed using MultiQuant 3.0.3 software. Chromatographic peaks of the analytes detected in different samples were integrated and corrected by referencing the retention times and peak profiles of the standards to ensure accurate qualification and quantification. The integration correction results for quantitative analysis of a randomly selected compound across different samples are presented in Supplementary Figure S5. Carotenoid standard solutions were prepared at concentrations of 0.001, 0.005, 0.01, 0.05, 0.1, 0.5, 1, 5, 10, and 25 μg/mL. The chromatographic peak intensity data corresponding to the quantitative signals of each concentration were obtained. Standard curves for different compounds were constructed by plotting the standard concentration (x-axis) against the peak area (y-axis). The linear equations and correlation coefficients of the standard curves for the detected compounds are provided in Supplementary Table S3.

**Figure 8:**
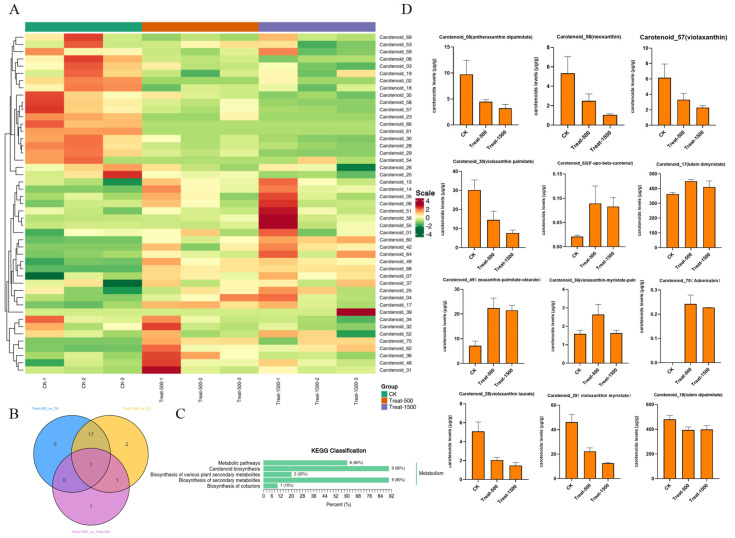
Metabolite clustering analysis and metabolite content. (**A**) Overall Metabolic clustering diagram of samples; (**B**) Venn diagram of differential metabolites in each treatment group; (**C**) KEGG classification map of differential metabolites (Treat-1500_vs_Ck); (**D**) Contents of various metabolites in the metabolome.

Among these, 47 metabolites were actually detected in the samples, including 40 lutein-type metabolites and 7 carotenoid-type metabolites. The data were subsequently processed with UV (unit variance scaling), and hierarchical clustering analysis was performed to examine the accumulation patterns of metabolites across different samples. A cluster heatmap was generated using R programming scripts (Figure 8A). The heatmap revealed 23 up-regulated metabolites and 24 down-regulated metabolites. By screening for differential metabolites with |fold change| > 2 and VIP > 1.0, a total of 20 differential metabolites were identified across the groups, including 9 up-regulated and 11 down-regulated metabolites. Among these, one common differential metabolite, neoxanthin (Carotenoid_58), a lutein-type metabolite, was found to be down-regulated across all groups (Figure 8B). The KEGG annotation results of significantly differential metabolites were categorized according to pathway types in KEGG. In the Treat_vs_CK KEGG pathway classification, Carotenoid biosynthesis and Biosynthesis of secondary metabolites accounted for the highest proportions, each representing 90% (Figure 8C).

In the Treat-500_vs_CK comparison, 18 differential metabolites were identified, including antheraxanthin dipalmitate (Carotenoid_08), α-cryptoxanthin (Carotenoid_64), β-cryptoxanthin (Carotenoid_60), neoxanthin (Carotenoid_58), 8′-apo-beta-carotenal (Carotenoid_62), violaxanthin laurate (Carotenoid_28), violaxanthin palmitate (Carotenoid_30), zeaxanthin-palmitate-stearate (Carotenoid_49), violaxanthin myristate (Carotenoid_29), β-citraurin (Carotenoid_68), adonirubin (Carotenoid_70), zeaxanthin-caprate-laurate (Carotenoid_42), rubixanthin caprate (Carotenoid_23), β-cryptoxanthin oleate (Carotenoid_54), canthaxanthin (Carotenoid_66), and astaxanthin (Carotenoid_61), which belong to the xanthophyll class. Additionally, ε-carotene (Carotenoid_07) and lycopene (Carotenoid_02) are carotenoids. Among these, α-cryptoxanthin (Carotenoid_64), β-cryptoxanthin (Carotenoid_60), 8′-apo-beta-carotenal (Carotenoid_62), zeaxanthin-palmitate-stearate (Carotenoid_49), β-citraurin (Carotenoid_68), adonirubin (Carotenoid_70), zeaxanthin-caprate-laurate (Carotenoid_42), and ε-carotene (Carotenoid_07) were up-regulated metabolites, while the remaining 10 were down-regulated.

In the CK_vs_Treat-500_vs_Treat-1500 comparison, 17 differential metabolites were identified, of which 16 were xanthophylls and one was a carotenoid. The changes in metabolite content are shown in Figure 8D. Among these, the most abundant metabolites were Carotenoid_17 (lutein dimyristate) and Carotenoid_18 (lutein dipalmitate), both of which are lutein esters. The content trend of Carotenoid_17 (lutein dimyristate) was consistent with the lutein content trend measured in earlier physiological experiments.

### 3.7. Integrated Analysis of Transcriptome and Metabolome

**Figure 9:**
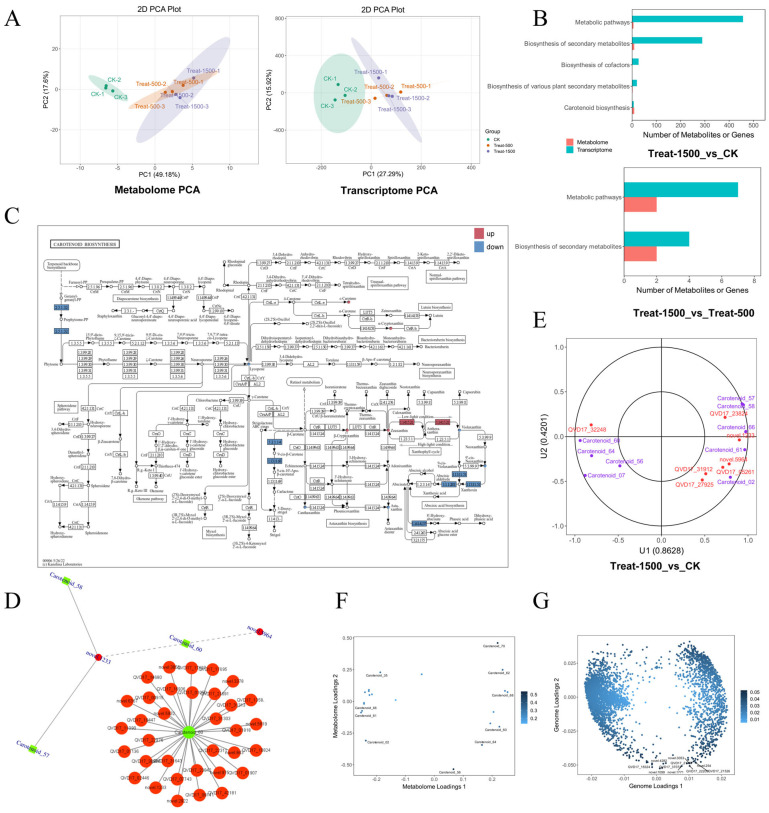
Transcriptional and metabolic joint analysis diagram. (**A**) Principal Component Analysis (PCA) of the transcriptome and metabolome; (**B**) KEGG enrichment analysis bar chart; (**C**) KEGG pathway diagrams co-mapped by differential metabolites and differential genes (Carotenoid biosynthesis pathway); (**D**) Network diagram of metabolite—gene correlations; (**E**) canonical correlation analysis; (**F**) O2PLS analysis of metabolite loadings plot; (**G**) O2PLS analysis of gene loadings plot.

In the integrated transcriptome and metabolome analysis, principal component analysis (PCA) of both datasets revealed substantial differences between the low-light and high-light conditions, whereas differences between Treat500 and Treat1500 were minimal (Figure 9A). This pattern was similarly reflected in KEGG enrichment bar plots, where the number and variety of enriched metabolites and differentially expressed genes in the Treat-1500_vs_CK comparison considerably exceeded those in the Treat-1500_vs_Treat-500 comparison. For the Treat-1500_vs_CK group, metabolites were primarily enriched in Metabolic pathways, Carotenoid biosynthesis, and Biosynthesis of secondary metabolites. By contrast, the Treat-1500_vs_Treat-500 comparison showed only a small number of differentially expressed genes and metabolites enriched in Metabolic pathways and Biosynthesis of secondary metabolites (Figure 9B).

Within the Carotenoid biosynthesis pathway under high-light conditions, α-Cryptoxanthin in the Lutein biosynthesis branch was up-regulated. Similarly, the xanthophyll metabolites Zeaxanthin and β-Cryptoxanthin also exhibited up-regulation. Conversely, the *PSY* gene (EC:2.5.1.32), located upstream in the Carotenoid biosynthesis pathway, was down-regulated (Figure 9C).

Correlation analysis between differentially expressed genes and differential metabolites was subsequently performed. Results with an absolute Pearson correlation coefficient greater than 0.8 and a *p*-value less than 0.05 were selected from the overall analysis and visualized in a network diagram (Figure 9D). This analysis revealed that β-cryptoxanthin (Carotenoid_60), neoxanthin (Carotenoid_58), and violaxanthin (Carotenoid_57) were collectively correlated with the novel.1233 gene, which encodes an Enoyl-(Acyl carrier protein) reductase (Figure 9D). The metabolite β-cryptoxanthin (Carotenoid_60) was associated with 755 genes; a subset of genes with stronger associations is displayed in Figure 9D, including genes for NAC transcription factors, CCHC zinc finger proteins, and MADS-box transcription factor FUL3, suggesting their potential indirect involvement in the xanthophyll metabolic pathway

The small circles and boxes in the diagram represent metabolites and genes, respectively. Red represents the up-regulated state of genes or metabolites, blue represents the down-regulated state of genes or metabolites, and yellow represents genes or metabolites that contain both up-regulated and down-regulated states. (Figure 9C).

Canonical Correlation Analysis (CCA) indicated a strong association between the QVD17_27925 (*CCD7*) gene, the QVD17_05261 (*CCD4-like*) gene, and the metabolites Astaxanthin (Carotenoid_61) and Lycopene (Carotenoid_61). Both genes belong to the Carotenoid Cleavage Dioxygenase (CCD) family and represent key enzymes in carotenoid catabolism (Figure 9E).

To further explore the overall relationship between the metabolome and genome, an O2PLS analysis was conducted. Figure 9F displays the significant metabolome variables with a substantial impact on the genome, showing the top 10 most influential metabolites. Among these, Lycopene (Carotenoid_61) was the only carotenoid, while the remaining nine were xanthophylls. Figure 9G presents the significant genomic variables that markedly influence the metabolome, with darker colors indicating a stronger degree of association.

## 4. Discussion

### 4.1. The Full-Flowering Stage (S3) Is a Critical Period for Lutein Accumulation

The lutein content in marigold petals peaked at the full-bloom stage (S3) (Figure 1E), identifying this stage as the optimal harvesting period for marigold pigment to maximize lutein yield. Analysis of key genes in the lutein synthesis pathway showed that upstream synthesis genes, including *TeGGPS*, *TePSY*, and *TeZDS*, were most highly expressed at the bud stage (S0) and subsequently declined (Figure 2B). This pattern aligns with findings in other plant species, where carotenoid biosynthetic structures are established through elevated gene expression prior to flowering [28]. Conversely, the downstream gene *TeCRITSO* and the degradation gene *TeCCD4* reached their highest expression levels at the petal coloration stage (S1) (Figure 2B), indicating active carotenoid turnover and catabolism during early flower opening. The distinct temporal expression patterns of synthesis and degradation genes collectively fine-tune lutein levels, which ultimately peak once a dynamic equilibrium is established at the full-flowering stage.

### 4.2. 500 μmol·m^−2^·s^−1^ Is the Optimal Condition for Promoting Lutein Accumulation

Light intensity serves as a key environmental factor regulating plant secondary metabolism. Our findings demonstrate that light intensity markedly influences the growth cycle, floral organ development, and lutein accumulation in marigolds. Compared with low light conditions, increased light intensity substantially shortened the growth period and enlarged flower diameter (Figure 3B,C), consistent with the established role of photosynthesis in supplying energy and carbon skeletons for plant growth and development [29].

These findings indicate that light intensity is a key environmental factor regulating the flowering process in marigolds. As light intensity increased from ≈30 μmol·m^−2^·s^−1^ to 1500 μmol·m^−2^·s^−1^, the time required for marigolds to reach the bud stage was significantly shortened, and the overall flowering process was accelerated. Under high light conditions (≈1500 μmol·m^−2^·s^−1^), the growth cycle was approximately 26 days shorter compared to that under low light conditions (≈30 μmol·m^−2^·s^−1^), accompanied by a significant increase in flower diameter (Figure 3B,C). This phenomenon aligns with the fundamental principle that photosynthesis provides energy and carbon sources for plant growth and development [30]. Accelerated development under higher irradiance has been documented in other species and is often linked to enhanced photosynthetic capacity and altered phytohormone signaling [29].

Regarding the molecular mechanism by which light intensity regulates floral organ development, studies in rose (*Rosa* sp.) offer relevant insights. Research by Wang Changquan’s team untangled the RcphyB–RcOST1L–RcPIF4 module, which mediates light intensity–responsive flowering in rose: under high light, RcphyB recruits RcOST1L and promotes its nuclear accumulation, subsequently reducing the stability of the phytochrome-interacting factor RcPIF4, ultimately accelerating flowering [31]. It is hypothesized that a similar regulatory mechanism may exist in marigolds. High light intensity may positively regulate the expression of downstream genes related to floral organ development by modulating the activity and stability of key components (such as *PhyB* or *PIF4* homologs) within its endogenous light signaling pathway, thereby promoting flowering and increasing flower diameter.

Notably, lutein content did not rise continuously with increasing light intensity, but reached a maximum at ≈500 μmol m^−2^·s^−1^ (Figure 3D). This pattern aligns with observations in crops such as lettuce, where moderate light elevation enhances carotenoid synthesis [32]. However, further increasing light intensity to ≈1500 μmol m^−2^·s^−1^ led to a reduction in lutein content. This decrease likely stems from high light-induced photoinhibition, which damages the photosynthetic apparatus and/or diverts carotenoids—especially lutein-cycle components like zeaxanthin—toward photoprotection, thereby lowering steady-state lutein accumulation [33]. Supporting this explanation, transcriptomic data revealed the downregulation of photosynthesis- and light-harvesting-related genes under high light conditions (Figure 6B,C).

### 4.3. Integrated Multi-Omics Analysis Untangles the Complex Network of Light Intensity in Regulating Lutein Metabolism

Integrated transcriptomic and metabolomic data enabled the construction of a molecular network that governs light intensity–dependent lutein metabolism in marigold. KEGG and GO enrichment analyses identified metabolic pathways, secondary metabolite biosynthesis, and plant hormone signal transduction as central processes influenced by light intensity (Figure 5 and Figure 6). Notably, under high light conditions, expression of the upstream carotenoid biosynthesis gene *TePSY* was down-regulated (Figure 9C), possibly reflecting a feedback mechanism to limit excessive intermediate accumulation under heightened energy availability. Metabolomic profiling, however, showed increased levels of downstream lutein metabolites, including α-cryptoxanthin, zeaxanthin, and β-cryptoxanthin, under strong light (Figure 9C,D). This apparent discrepancy may reflect the temporally regulated and cumulative nature of carotenoid biosynthesis. Our developmental stage data (Figure 2B) show that upstream biosynthetic genes, including *TePSY* and *TeGGPS*, were most highly expressed during the early bud stages (S0, S1). This expression pattern suggests that a substantial pool of carotenoid precursors, such as phytoene and lycopene, is synthesized prior to anthesis. These precursors accumulate in plastids, forming a substrate reservoir that supports the subsequent burst of metabolite accumulation during later floral developmental stages like the full-bloom stage (S3). Consequently, the high xanthophyll content observed at S3 likely results mainly from the continuous conversion of pre-existing intermediate pools, rather than from intensive de novo synthesis specifically at S3. Within this framework, the downregulation of *TePSY* may reflect cellular feedback inhibition to optimize resource allocation and prevent the energy expenditure associated with excessive synthesis. The dissociation between transcript levels and metabolite accumulation further implies the involvement of post-transcriptional or post-translational regulation, or enhanced xanthophyll esterification that facilitates storage [34].

Lutein esters such as lutein dimyristate and lutein dipalmitate were identified as the predominant storage forms in petals, with accumulation patterns mirroring total lutein content (Figure 8D). Correlation network analysis revealed strong connections among transcription factors (e.g., NAC, MADS-box FUL3), carotenoid cleavage dioxygenase genes (*CCD4-like*, *CCD7*), and key lutein metabolites, including β-cryptoxanthin, astaxanthin, and lycopene (Figure 9D,E). Upregulation of *CCD* genes may facilitate degradation of specific carotenoids, reshaping the metabolic landscape, particularly under high light [13]. Additionally, several light-responsive transcription factor families—such as bHLH, MYB, NAC, and WRKY—were identified, with the upregulation of *WRKY24* representing a novel observation (Figure 7C,D). Although *WRKY24* has not been previously implicated in carotenoid metabolism, WRKY family members are recognized for integrating environmental cues and modulating secondary metabolism [35].

## 5. Conclusions

This study documented organ initiation and development in marigold flowers, established a classification of developmental stages, and identified the S3 stage as the optimal harvest period according to lutein content measured across these stages. Lutein content reached its maximum under a light intensity of ≈500 μmol m^−2^·s^−1^. Upstream genes in the lutein synthesis pathway, including *TeGGPS*, *TePSY*, and *TeZDS*, exhibited high expression during the bud stage, whereas downstream modification genes and the cleavage enzyme gene *TeCCD4* responded more strongly at flowering. Light dynamically modulates the balance between synthesis and degradation pathways by activating transcription factor families such as bHLH, MYB, NAC, and WRKY. Metabolomic profiling identified lutein esters, principally lutein dimyristate and lutein dipalmitate, as the dominant accumulated forms, with their levels showing a significant positive correlation with light intensity. Integrated transcriptomic and metabolomic analyses indicated significant enrichment of differential metabolites and genes when comparing weak and strong light conditions. However, increasing light intensity beyond a certain threshold did not further enhance carotenoid accumulation. Physiological data confirmed that 500 μmol m^−2^·s^−1^ represents the optimal light intensity for marigold growth.

These findings suggest several directions for future research, beginning with the functional characterization of identified transcription factors such as bHLH, MYB, NAC, and WRKY. In marigolds, the enzymes responsible for the key esterification of lutein remain unknown. Identifying these lutein acyltransferases would significantly advance our understanding of the final steps in lutein stabilization and storage.

This study reveals how light precisely regulates lutein accumulation through coordinated synthesis, esterification, and degradation pathways. It thereby establishes a theoretical basis for improving lutein production in marigold via light-mediated cultivation and offers fundamental data on the light intensity–responsive patterns that govern lutein accumulation.

## Data Availability

The original contributions presented in this study are included in the article/Supplementary Material. Further inquiries can be directed to the corresponding author.

## Supplementary Materials

The following supporting information can be downloaded at: https://www.mdpi.com/article/10.3390/genes16111350/s1.

## References

[1] Hoshi Y., Praphruet R., Hironaka K. (2019). Genome Size Determination and Chromosome Characterization of Two Marigold Species (*Asteraceae*). Cytologia.

[2] Zhang H.L., Cong R.C., Wang M.L., Dong A.X., Xin H.B., Yi M.F., Guo H. (2018). Development of SSR Molecular Markers Based on Transcriptome Sequencing of *Tagetes erecta*. Acta Hortic. Sin..

[3] Piccaglia R., Marotti M., Grandi S. (1998). Lutein and Lutein Ester Content in Different Types of *Tagetes patula* and *T. erecta*. Ind. Crops Prod..

[4] Xin H.B., Ji F.F., Wu J., Zhang S., Yi C., Zhao S., Cong R., Zhao L., Zhang H., Zhang Z. (2023). Chromosome-Scale Genome Assembly of Marigold (*Tagetes erecta* L.): An Ornamental Plant and Feedstock for Industrial Lutein Production. Hortic. Plant J..

[5] Berman J., Zorrilla-Lopez U., Farre G., Zhu C., Sandmann G., Twyman R.M., Capell T., Christou P. (2015). Nutritionally Important Carotenoids as Consumer Products. Phytochem. Rev..

[6] Mares J. (2016). Lutein and Zeaxanthin Isomers in Eye Health and Disease. Annu. Rev. Nutr..

[7] Mitri K., Shegokar R., Gohla S., Anselmi C., Müller R.H. (2011). Lutein Nanocrystals as Antioxidant Formulation for Oral and Dermal Delivery. Int. J. Pharm..

[8] Gombač Z., Črnivec I.G.O., Skrt M., Istenič K., Knafelj A.K., Pravst I., Ulrih N.P. (2021). Stabilisation of Lutein and Lutein Esters with Polyoxyethylene Sorbitan Monooleate, Medium-Chain Triglyceride Oil and Lecithin. Foods.

[9] Moehs C.P., Tian L., Osteryoung K.W., Dellapenna D. (2001). Analysis of carotenoid biosynthetic gene expression during marigold petal development. Plant Mol. Biol..

[10] Colasuonno P., Lozito M.L., Marcotuli I., Nigro D., Giancaspro A., Mangini G., De Vita P., Mastrangelo A.M., Pecchioni N., Houston K. (2017). The Carotenoid Biosynthetic and Catabolic Genes in Wheat and Their Association with Yellow Pigments. BMC Genom..

[11] Bruno M., Beyer P., Al-Babili S. (2015). The Potato Carotenoid Cleavage Dioxygenase 4 Catalyzes a Single Cleavage of β-Ionone Ring-Containing Carotenes and Non-Epoxidated Xanthophylls. Arch. Biochem. Biophys..

[12] Giorio G., Yildirim A., Stigliani A.L., D’Ambrosio C. (2013). Elevation of Lutein Content in Tomato: A Biochemical Tug-of-War Between Lycopene Cyclases. Metab. Eng..

[13] Auldridge M.E., Block A., Vogel J.T., Dabney-Smith C., Mila I., Bouzayen M., Magallanes-Lundback M., DellaPenna D., McCarty D.R., Klee H.J. (2006). Characterization of Three Members of the Arabidopsis Carotenoid Cleavage Dioxygenase Family Demonstrates the Divergent Roles of This Multifunctional Enzyme Family. Plant J..

[14] Zhang H., Zhang S., Zhang H., Chen X., Liang F., Qin H., Zhang Y., Cong R., Xin H., Zhang Z. (2020). Carotenoid Metabolite and Transcriptome Dynamics Underlying Flower Color in Marigold (Tagetes erecta L.). Sci. Rep..

[15] Pinnola A., Gerotto C., Morosinotto T., Bassi R., Alboresi A. (2013). Zeaxanthin Binds to Light-Harvesting Complex Stress-Related Protein to Enhance Nonphotochemical Quenching in *Physcomitrella* patens. Plant Cell.

[16] Stange C. (2016). Carotenoids in Nature. Subcell. Biochem..

[17] Ma R.J., Zhao X.R., Xie Y.P., Ho S.H., Chen J.F. (2019). Enhancing Lutein Productivity of *Chlamydomonas* sp. via High-Intensity Light Exposure with Corresponding Carotenogenic Genes Expression Profiles. Bioresour. Technol..

[18] Schüler L.M., Santos T., Pereira H., Duarte P., Gangadhar K.N., Florindo C., Schulze P.S.C., Barreira L., Varela J.C.S. (2020). Improved Production of Lutein and β-Carotene by Thermal and Light Intensity Upshifts in the Marine Microalga *Tetraselmis* sp. CTP4. Algal Res..

[19] Das P.R., Del Moro D.S., Givens S.R., Armstrong S.P., Walters K.J. (2024). Propagation Light Intensity Influences Yield, Morphology, and Phytochemistry of Purple-Leaf Butterhead Lettuce (*Lactuca sativa*). J. Agric. Food Res..

[20] Akemi O., Masaya K., Takehiko S., Kenji N., Sanae K., Masayasu N. (2019). Molecular Basis of Carotenoid Accumulation in Horticultural Crops. Hortic. J..

[21] Rodrigues D.B., Mercadante A.Z., Mariutti L.R.B. (2019). Marigold carotenoids: Much more than lutein esters. Food Res. Int..

[22] Feng G.D. (2019). Transcriptome Analysis and Molecular Mechanism of Carotenoid Synthesis Pathway of Marigold. PhD. Thesis.

[23] Livak K.J., Schmittgen T.D. (2001). Analysis of Relative Gene Expression Data Using Real-Time Quantitative PCR and the 2^−ΔΔCT^ Method. Methods.

[24] Krinsky N.I., Mayne S.T., Sies H. (2004). Carotenoids in Health and Disease.

[25] Bartley E.G., Scolnik P.A. (1995). Plant Carotenoids: Pigments for Photoprotection, Visual Attraction and Human Health. Plant Cell.

[26] Inbaraj B.S., Lu H., Hing C.F., Wu C.F., Lin C.L., Chen B.H. (2008). Determination of Carotenoids and Their Esters in Fruits of *Lycium barbarum* Linnaeus by HPLC-DAD-APCI-MS. J. Pharm. Biomed. Anal..

[27] Geyer R., Peacock A.D., White D.C., Lytle C., Van G.J. (2004). Atmospheric Pressure Chemical Ionization and Atmospheric Pressure Photoionization for Simultaneous Mass Spectrometric Analysis of Microbial Respiratory Ubiquinones and Menaquinones. J. Mass Spectrom..

[28] Nisar N., Li L., Lu S., Khin N.C., Pogson B.J. (2015). Carotenoid Metabolism in Plants. Mol. Plant.

[29] Del Villar-Martínez A.A., García-Saucedo P.A., Carabez-Trejo A., Cruz-Hernández A., Paredes-Lópeza O. (2005). Carotenogenic gene expression and ultrastructural changes during development in marigold. J. Plant Physiol..

[30] Li Y., Kubota C. (2009). Effects of Supplemental Light Quality on Growth and Phytochemicals of Baby Leaf Lettuce. Environ. Exp. Bot..

[31] Sun J., Liu H., Wang W., Fan C., Yuan G., Zhou R., Lu J., Liu J., Wang C. (2024). RcOST1L phosphorylates RcPIF4 for proteasomal degradation to promote flowering in rose. New Phytol..

[32] Maragò E., Michelozzi M., Calamai L., Camangi F., Sebastiani L. (2016). Antioxidant properties, sensory characteristics and volatile compounds profile of apple juices from ancient Tuscany (Italy) apple varieties. Eur. J. Hortic. Sci..

[33] Demmig-Adams B., Adams III W.W. (1996). The Role of Xanthophyll Cycle Carotenoids in the Protection of Photosynthesis. Trends Plant Sci..

[34] Sun T., Yuan H., Cao H., Yazdani M., Tadmor Y., Li L. (2018). Carotenoid Metabolism in Plants: The Role of Plastids. Mol. Plant.

[35] Phukan U.J., Jeena G.S., Shukla R.K. (2016). WRKY Transcription Factors: Molecular Regulation and Stress Responses in Plants. Front. Plant Sci..

