# Supplementary figures and images for: Integrated Transcriptomic and Metabolomic Analyses of the Response of Lutein Accumulation in Marigold Petals to Light Intensity
Source: Genes (Basel). 2025 Nov 9;16(11):1350. doi: 10.3390/genes16111350 (PMC12652989; doi:10.3390/genes16111350)

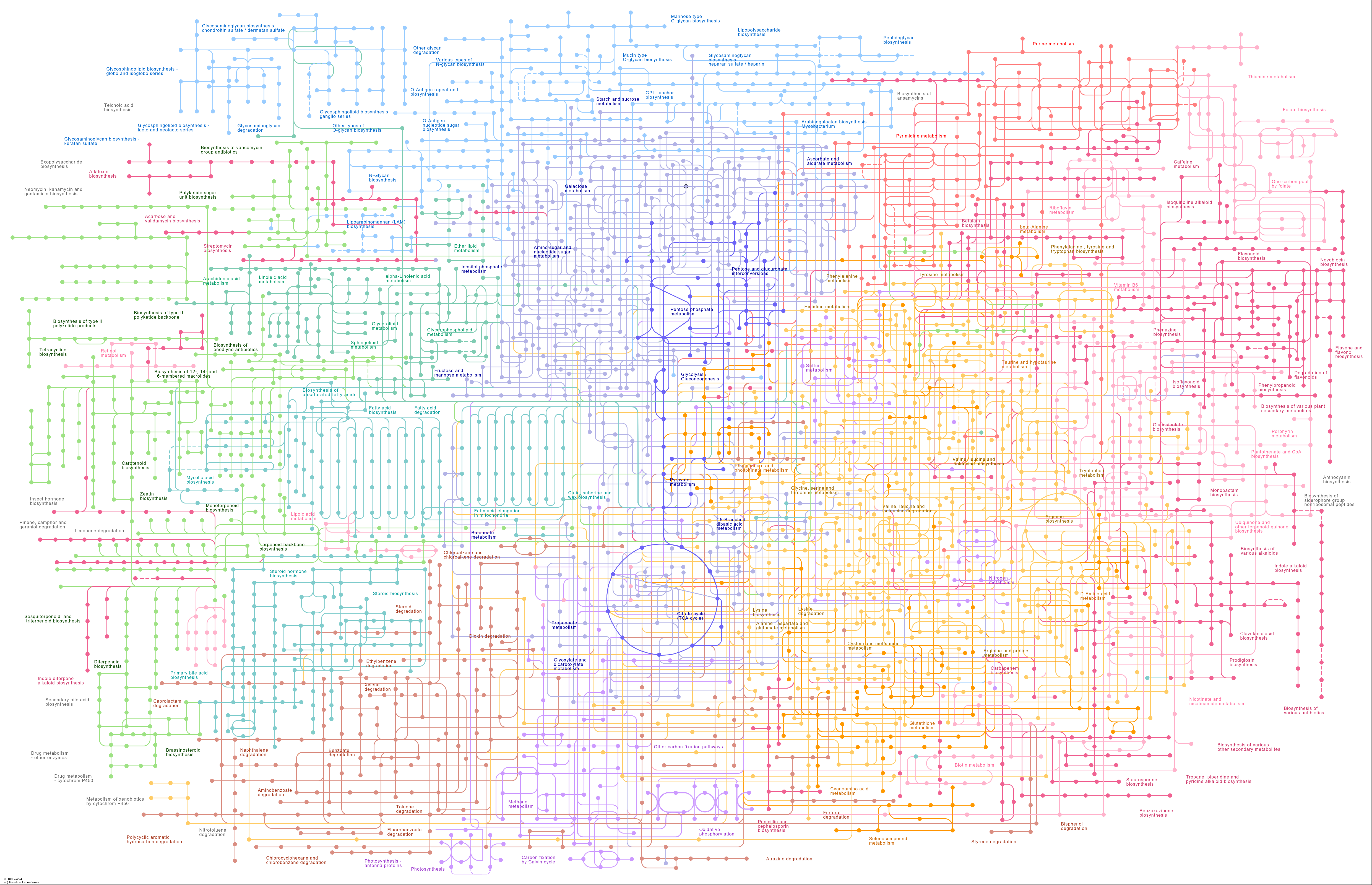

Supplement: Supplementary file 1 [file genes-16-01350-s001.zip › Supplementary Figure S1-ko01100.png]

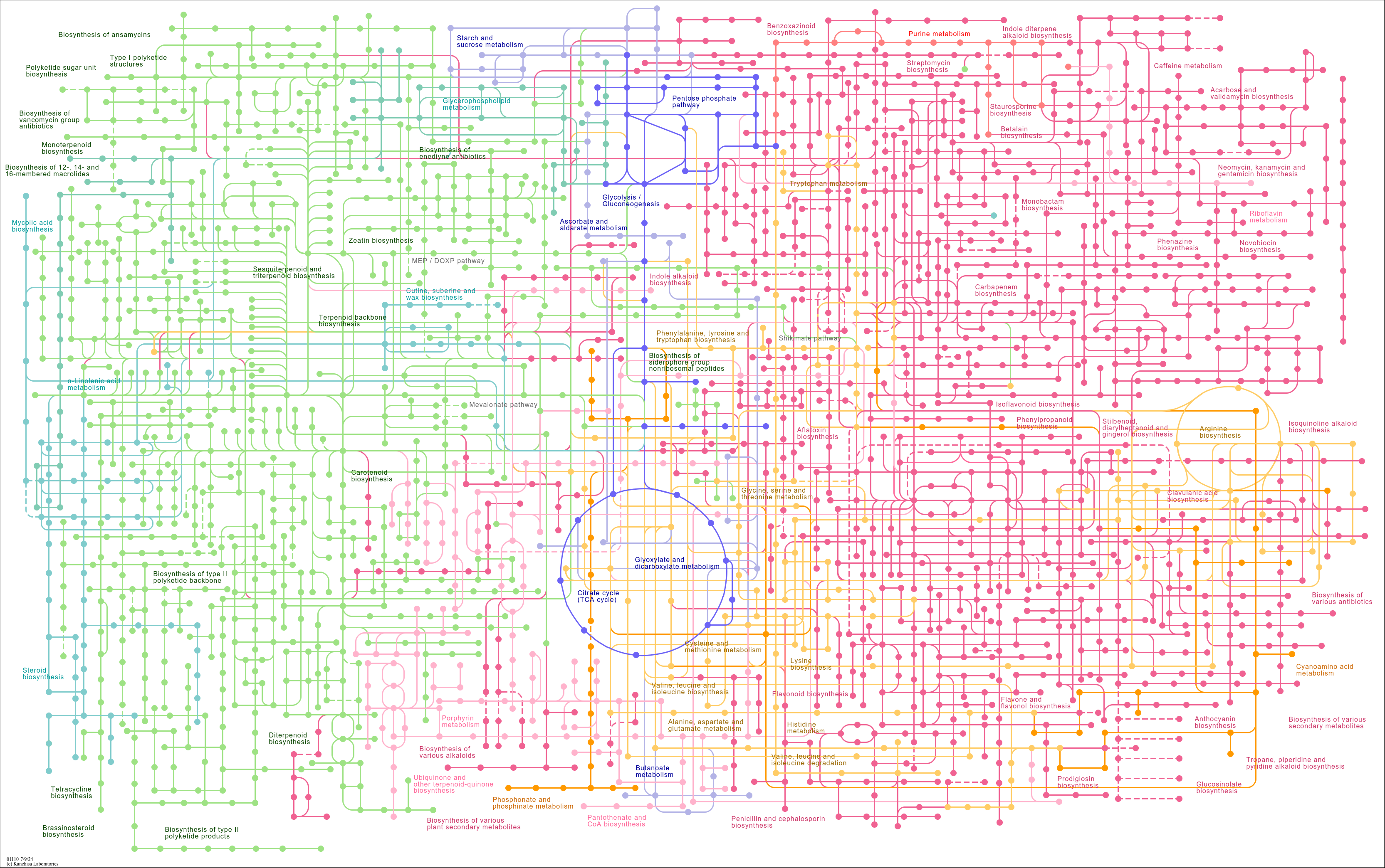

Supplement: Supplementary file 1 [file genes-16-01350-s001.zip › Supplementary Figure S2-Ko01110.png]

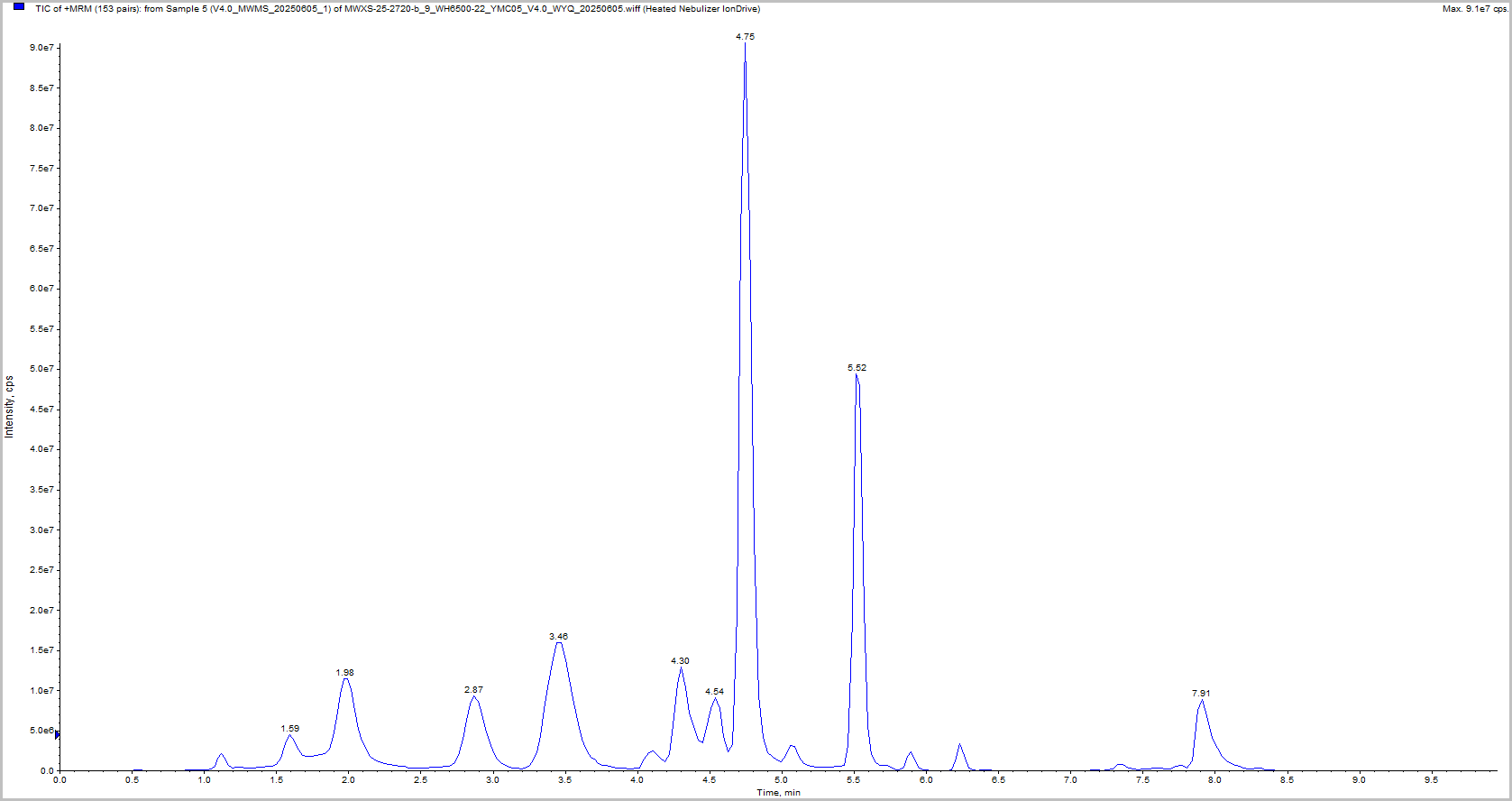

Supplement: Supplementary file 1 [file genes-16-01350-s001.zip › Supplementary Figure S3-_TIC.png]

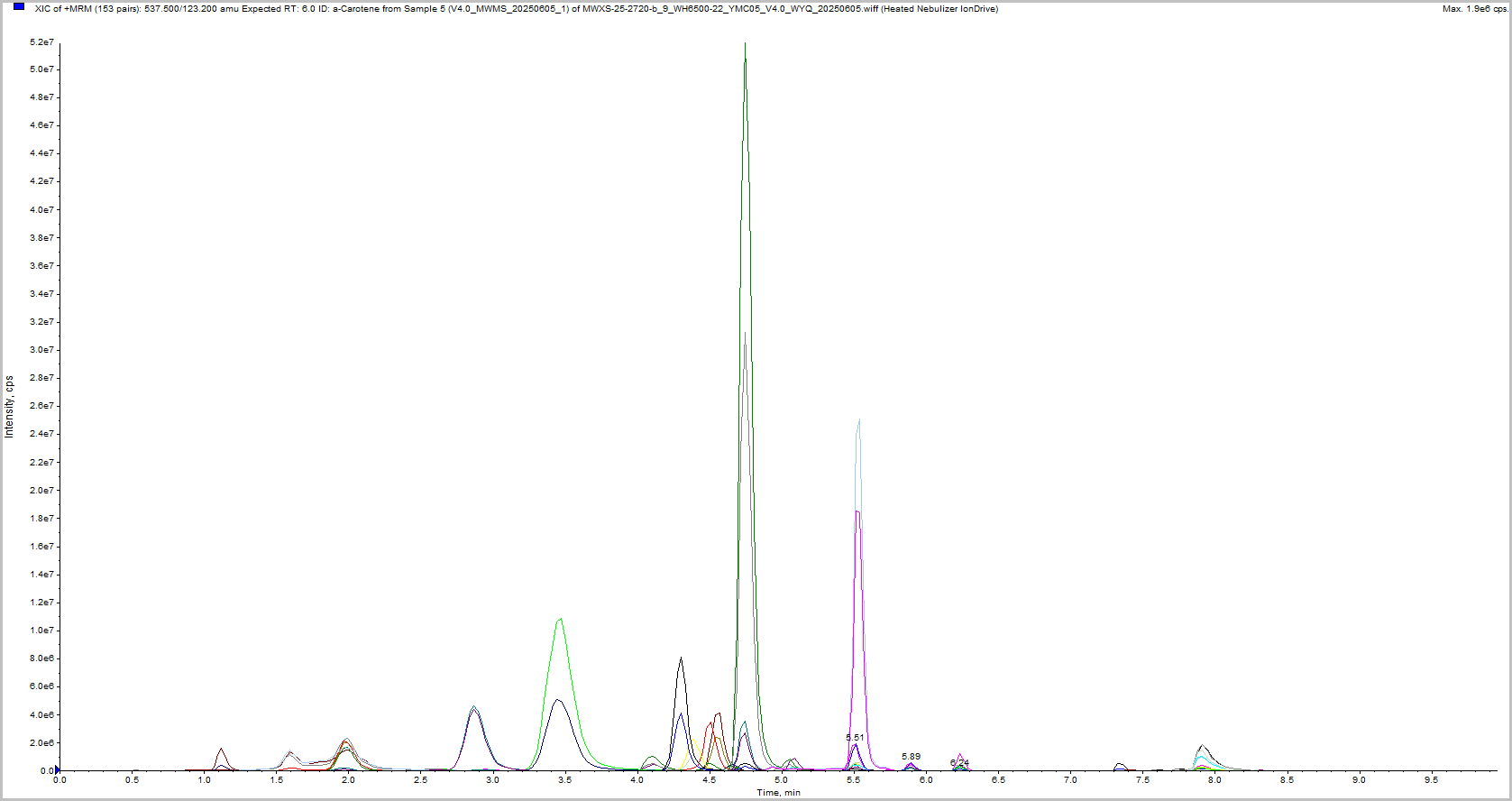

Supplement: Supplementary file 1 [file genes-16-01350-s001.zip › Supplementary Figure S4-XIC.png]

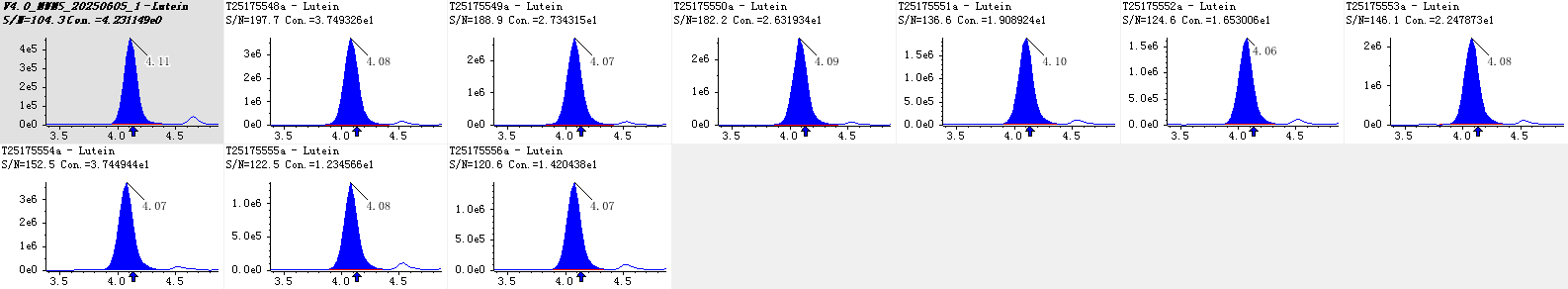

Supplement: Supplementary file 1 [file genes-16-01350-s001.zip › Supplementary Figure S5.png]
